# Identification of potential Akt activators: a ligand and structure-based computational approach

**DOI:** 10.1007/s11030-023-10671-1

**Published:** 2023-07-03

**Authors:** Harish B. Kumar, Suman Manandhar, Ekta Rathi, Shama Prasada Kabekkodu, Chetan Hasmukh Mehta, Usha Yogendra Nayak, Suvarna G. Kini, K. Sreedhara Ranganath Pai

**Affiliations:** 1 Department of Pharmacology, Manipal College of Pharmaceutical Sciences, Manipal Academy of Higher Education, Manipal, Karnataka 576104 India; 2 Department of Pharmaceutical Chemistry, Manipal College of Pharmaceutical Sciences, Manipal Academy of Higher Education, Manipal, Karnataka 576104 India; 3 Department of Cell and Molecular Biology, Manipal School of Life Sciences, Manipal Academy of Higher Education, Manipal, Karnataka 576104 India; 4 Department of Pharmaceutics, Manipal College of Pharmaceutical Sciences, Manipal Academy of Higher Education, Manipal, Karnataka 576104 India

**Keywords:** Pharmacophore modeling, Fingerprint-based 2D QSAR, Shape-based screening, Akt activator, Molecular dynamic simulation

## Abstract

The Akt pathway plays a significant role in various diseases like Alzheimer’s, Parkinson’s, and Diabetes. Akt is the central protein whose phosphorylation controls many downstream pathways. Binding of small molecules to the PH domain of Akt facilitates its phosphorylation in the cytoplasm and upregulates the Akt pathway. In the current study, to identify Akt activators, ligand-based approaches like 2D QSAR, shape, and pharmacophore-based screening were used, followed by structure-based approaches such as docking, MM-GBSA, ADME prediction, and MD simulation. The top twenty-five molecules from the Asinex gold platinum database found to be active in most 2D QSAR models were used for shape and pharmacophore-based screening. Later docking was performed using the PH domain of Akt1 (PDB: 1UNQ), and 197105, 261126, 253878, 256085, and 123435 were selected based on docking score and interaction with key residues, which were druggable and formed a stable protein–ligand complex. MD simulations of 261126 and 123435 showed better stability and interactions with key residues. To further investigate the SAR of 261126 and 123435, derivatives were downloaded from PubChem, and structure-based approaches were employed. MD simulation of derivatives 12289533, 12785801, 83824832, 102479045, and 6972939 was performed, in which 83824832 and 12289533 showed interaction with key residues for a longer duration of time, proving that they may act as Akt activators.

**Supplementary Information:**

The online version contains supplementary material available at 10.1007/s11030-023-10671-1.

## Introduction

Protein kinase B (Akt) is a serine/threonine kinase that lies at the nexus of survival and cell death pathways and is crucial in numerous cell signaling systems associated with cell growth, division, metabolism, angiogenesis, and apoptosis. Akt displays critical metabolic effects, including glucose uptake in fat and muscle cells or the subduing neuronal cell death. Various diseases, such as Diabetes, cardiovascular, and neurological disorders, correlate well with the derangement of Akt-regulated pathways.

The isoforms of Akt, known as Akt1, Akt2, and Akt3, are encoded by three separate genes; however, they all have a conserved structural domain composed of a kinase domain, an N-terminal pleckstrin homology domain (PH domain), and a C-terminal regulatory region containing a hydrophobic motif [1]. Akt isoforms are expressed differently in various tissues; the expression of Akt1 is ubiquitous, Akt2 is concentrated in organs that react to insulin, and Akt3 is substantially expressed in the brain and testes [2].

Akt pathway activation begins with ligand binding to cell surface receptor-like G-protein-coupled receptors (GPCRs), receptor tyrosine kinases (RTKs), and insulin receptor substrate (IRS), which leads to the phosphorylation of phosphoinositide 3-kinase (PI3K). Lipids are then phosphorylated by activated PI3K on the plasma membrane, leading to the formation of the secondary messenger phosphatidylinositol (3,4,5)-trisphosphate (PIP3) from phosphatidylinositol (3,4)-bisphosphate (PIP2). Akt is then recruited to the membrane by binding PIP3 to the PH domain of Akt, which brings about a conformational change required for phosphorylation by 3-Phosphoinositide-dependent kinase 1 (PDK1) at T308, and full activation is achieved by phosphorylation at S473 by mammalian target of rapamycin complex 2 (mTORC2). After activation of Akt, it acts on many downstream targets such as glycogen synthase kinase-3 beta (GSK-3β), Bcl-2 agonist of cell death (BAD), Forkhead, and mTOR pathways [3].

Akt1 has three domains: the PH domain (5–108), kinase (150–408), and AGC-kinase C (409–480) terminal. The PH domain has two orthogonal anti-parallel β-sheets formed by seven β-strands that are closed by an α-helix at the C-terminal at one end and by a β-barrel at the other. The β-barrel contains VL1–VL3 variable loops with different sequences and lengths. Phosphatidylinositol-(3,4,5)-triphosphate (PtdIns(3,4,5)P3) and phosphatidylinositol-(3,4)-bisphosphate (PtdIns(3,4)P2) head groups bind to these loops, forming a highly basic pocket. The Apo AktαPH domain forms a complex hydrogen-bonding network centered around ionic interactions between Lys 14, Glu 17, Asn 53, and Arg 86, as well as several water molecules. Upon binding, PtdIns(1,3,4,5)P4 disrupts the network of hydrogen bonds (H-bonds), resulting in the shift of Arg 86 (2.3 Å) towards 4-phosphate, Lys 14 (1.2 Å) towards 3- and 4-phosphates, and Arg 23 (6.2 Å) towards 1- and 3-phosphates. There is no change in the position of Asn 53, although it binds to the 3- and 4-phosphate groups. Acidic Glu 17 is repelled by phosphoinositide due to its negative charge, resulting in a conformational change of VL1 with backbone shifts at Tyr 18 (2.5 Å). In addition, Tyr 18 creates space for 5-phosphate by moving 5.0 Å away from the binding pocket, preventing steric hindrance. The movement of Glu 17 away from the binding pocket of the ligand enables the movement of Arg 86 towards 4-phosphate. The VL3 loop undergoes a considerable conformational change, moving up to 7.4 towards the phosphoinositide-binding pocket in the complex ligand bound compared to its position in the apo structure. The conformational shift caused by PtdIns(1,3,4,5)P4 binding could be attributed to the migration of Arg 86 at the base of VL3 towards 4-phosphate [4]. The structure and function of the kinase domain and AGC-kinase C-terminal are in the supplementary introduction.

Akt is a valuable therapeutic target because it regulates the Akt signaling pathway. The discovery of Akt activators using various strategies, such as high-throughput cell-based assays, has led to identifying activators such as SC79 [7]. Although it has shown promising activity in multiple diseases, such as Diabetes, Alzheimer’s disease, ischemia-reperfusion injury, and stroke, the molecule is relatively unstable in aqueous environments. Later, while studying the mechanism of action of many phytochemicals that help in Diabetes, Bai et al. identified more molecules that activate Akt, like baicalin [8], puerarin [9], chlorogenic acid [10], kaempferol-3-glucuronide [11], and quercetin-3-glucuronide [11]. To date, high-throughput virtual screening computational techniques have not been applied, possibly because of the lack of a crystal structure of full-domain Akt1 in the active conformation. Therefore, various ligand and structure-based computational tools were used for the first time to identify lead molecules that may act as Akt activators.

## Materials and methodology

Computational studies were performed using the Schrödinger suite version 2018–3 for fingerprint-based 2D quantitative structure–activity relationship (QSAR) and 2022–3 for all other studies (Schrödinger, LLC, New York) on the Ubuntu platform, using tools such as Ligprep, Canvas, Phase, shape screening, protein preparation wizard, GLIDE, WaterMap, and Desmond.

### Protein selection and preparation

The PH domain of Akt1 (PDB ID:1UNQ), which binds to inositol 1,3,4,5-tetrakisphosphate, is the only crystal structure available in which the PH domain of Akt1 is in its active conformation; therefore, the same PDB was used for this study. The crystal structure of 1UNQ was downloaded from the RCSB website (http://www.rcsb.org/) and processed using Protein Preparation Wizard [12]. First, missing hydrogen bonds and amino acid residues were added, and the ionization state of the het group was generated at pH 7.5 ± 0.2. In the second step, PROPKA H-bonds were assigned and optimized; subsequently, all water molecules in the protein structure were removed. Finally, the lowest energy level of the prepared protein structure was generated by minimizing the optimized potential for the liquid simulation (OPLS3e) force field.

### Ligand preparation and phase database creation

In this study, the Asinex gold platinum collection (305,168 molecules) from the Asinex database was downloaded, which contains a historical compound that provides diverse and cost-effective molecules with drug-like properties in accordance with Lipinski’s Rule of Five. After importing the molecules, they were optimized using the Ligprep tool [13] to obtain 3-dimensional structures with an optimized ionization state at pH 7.5 ± 0.2. The chirality of these molecules was determined using 3D structures, and energy minimization was performed using the OPLS3e force field.

For various ligandbased approaches, such as pharmacophorebased ligand screening and shapebased screening, phase database was created using prepared molecules without any ligand filter by generating a target number of conformers up to 800.

### Fingerprint-based 2D QSAR modeling

Canvas module (Schrodinger 2018-3) was used for the fingerprint-based 2D QSAR. A complex descriptor known as a fingerprint represents the two-dimensional structure of molecules. The different fingerprint arrangements generate a unique bit pattern for each molecule. The number of bits in the fingerprints is fixed and accounts for the connectivity between different structural fragments within the molecules. The bits represent the presence (1) and absence (0) of certain structural features in a bit string, and a hashed bit string is formed when all fragments are joined together into a bit string.

The active and inactive sets of molecules used in this study are listed in Supplementary Table S1. Seven different fingerprints were generated: radial, linear, molprint 2D, dendritic, atom triplet, atom pair, and topological torsion. These fingerprints were employed to develop the Bayer classification model by categorizing IC50 as active as one and inactive as zero, the Y variable was selected as the IC50, and the X variable was selected as a binary property. The test set of inactive molecules (SC7, SC12, SC16, SC18, SC23, and SC28) was kept constant; however, the active test set was changed, as shown in Table 1, and the remaining ligand was used in the training set. After building the model, it was tested, and the model with 0 incorrect predictions was used to screen databases after generating binary properties.

**Table 1:**
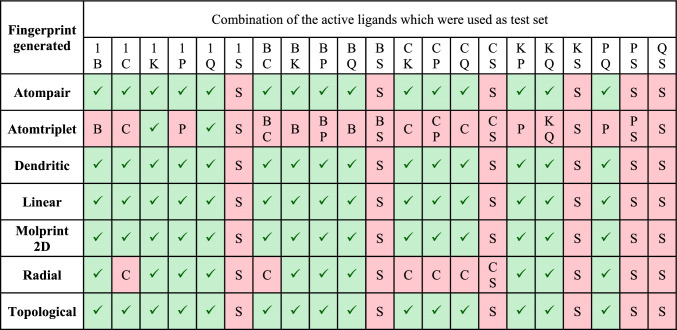
List of models built using different combinations of active ligands in the test set where 1—inositol 1,3,4,5-tetrakisphosphate, B—baicalin, C—chlorogenic acid, P—puerarin, K—kaempferol-3-O-glucuronide, Q—quercetin-3-O-glucuronide; Green color denotes models in which they were no incorrect prediction and red color models with wrong prediction

### Shape-based virtual screening

For shape screening [14], known Akt activators such as inositol 1,3,4,5-tetrakisphosphate, chlorogenic acid, puerarin, baicalin, SC79, kaempferol-3-glucuronide, and quercetin-3-glucuronide were used as a template to screen the phase database using pharmacophore-type volume scoring.

This approach uses phase feature definitions to map pharmacophore sites to a structure, with each site represented by a two-angstrom hard sphere. This technique does not imply any specific pharmacophore model, as all sites in each structure are encoded into the shape, not just those essential for binding to a particular target. Shape screening identifies many triplet pairs with similar geometries and local environments in the template and database structures. It then superimposes them based on the least-squares alignment of each pair of triplets using the triplet-based alignment; the superposition with the highest shape similarity is refined by realigning additional pairs of atoms/sites within 0.5 Ǻ of each other.

### Pharmacophore modeling and virtual screening

### Protein–ligand complex-based pharmacophore

PDB 1UNQ contains the PH domain of Akt1 bound to inositol 1,3,4,5-tetrakisphosphate; therefore, the prepared protein–ligand complex was used for e-pharmacophore [15] generation using the phase module. The e-pharmacophore generates a hypothesis based on the complementarity of receptor and ligand features. The automatic mode was used, in which Glide XP scoring terms were used to determine which features contributed the most to the binding.

### Multiple ligand-based pharmacophores

For multiple ligand-based pharmacophores, the phase program identified the pharmacophore features of each ligand. Common properties are sought to satisfy the requirements for their locations and orientations to create pharmacophore hypotheses. The hypotheses were scored based on geometric alignment and their ability to retrieve actives from a set of decoys and can be penalized to match inactive molecules. To develop the hypothesis for Akt1 activators, molecules that bind to the PH domain of Akt1, such as inositol 1,3,4,5-tetrakisphosphate, chlorogenic acid, puerarin, baicalin, SC79, kaempferol-3-glucuronide, and quercetin-3-glucuronide were used. The best fit and common features method was used, the target number of conformers for molecules was set at 800, and the hypothesis matched at least 80% of actives and had 4-5 pharmacophore features such as hydrogen bond donor, hydrogen bond acceptor, aromatic ring, negative ionic, positive ionic, and hydrophobic.

The phase database was screened using pharmacophore-based models in the Schrödinger PHASE module to find Akt1 activators with the desired chemical properties. For multiple ligand-based pharmacophores, compounds had to match at least four sites; for the protein–ligand complex-based e-pharmacophore hypothesis, the minimum match requirement was three sites. The phase fitness score, which evaluates how well compounds match the chemical properties of the pharmacophore sites based on vector alignments, volume terms, and root mean square deviation (RMSD) site matching, was used to rank the final hits from virtual screening.

### Molecular docking and binding free energy calculation

After performing various ligand-based approaches, such as shape-based screening, pharmacophore-based screening, and fingerprint-based 2D QSAR, a list of molecules that were found to be active using most models was created and considered for molecular docking.

The Glide module was used for molecular docking [16], and a grid was generated centroid to the inositol 1,3,4,5-tetrakisphosphate of 20 Å size. Using the generated receptor grid, the inbound ligand was redocked to validate the docking protocol by calculating the RMSD of the pose generated after docking compared to the pose in the crystal structure. After validation, the top twenty-five molecules were docked using extra precision (XP) docking. The docked molecules were then analyzed for their interaction patterns and docking score. The top five molecules obtained from molecular docking were selected for molecular mechanics generalized born surface area (MM-GBSA), which provides binding energy to the protein–ligand complex. The prime MM-GBSA module was used to determine the ligand-binding energies and ligand-strain energies for the top five molecules shortlisted in the docking studies. The OPLS3e force field and VSGB solvent model were used, while the molecules and receptors were obtained from the project table and workspace, respectively. Since the MM-GBSA binding energies are approximate free energies of binding, a lower value (kcal/mol) indicates stronger binding.

### Predicted pharmacokinetic property

The QikProp module was used to predict the absorption, distribution, metabolism, and excretion (ADME) characteristics of the selected top five molecules. To assess the drug-likeness of the chosen molecules, several properties and descriptors were predicted which includes molecular weight, predicted central nervous system activity, predicted aqueous solubility, predicted octanol/water partition coefficient, predicted apparent Caco-2 permeability, predicted brain/blood partition coefficient, QPlogKhsa,/ Lipinski rule of 5, and predicted human oral absorption.

### Molecular dynamics simulation

MD simulations [17, 18] were performed for all five hit molecules selected after extra precision docking. The Desmond module was used to generate periodic boundary conditions of orthorhombic shape with a size of 10 × 10 × 10 Å from the surface of the protein complex and solvated using the simple point charge water model (SPC) as a predefined solvent model. The system was neutralized by adding sodium and chloride ions to maintain the iso-osmotic condition and minimized for 100 ps using a 1.0 (kcal/mol/Å) convergence threshold and 2000 maximum iterations. In the final step, the minimized solvated system was used for running the MD simulation at the normal pressure (1.01 bar) and temperature (300 K) ensemble for 100 ns. After the simulation, a simulation interaction diagram was generated to analyze the MD results, such as the plot for the protein–ligand RMSD and protein–ligand contacts during the simulation.

## Result and discussion

### Fingerprint-based 2D QSAR modeling

After building various fingerprint-based 2D QSAR models, as discussed in Sect. 2.3, models that can accurately predict the active and inactive molecules in the test set were selected. Of the 147 models generated, only 87 could predict the test set of molecules without any error tabulated in Table 1 and were therefore used to predict the activity of 305,168 database molecules. After predicting the activity, the top twenty-five molecules found to be active using most models were enlisted in Table 2. Out of the top twenty-five molecules, only seven molecules, 109586, 115183, 125036, 142354, 197105, 253878, and 256085, were predicted to be active in all 87 models.

**Table 2:**
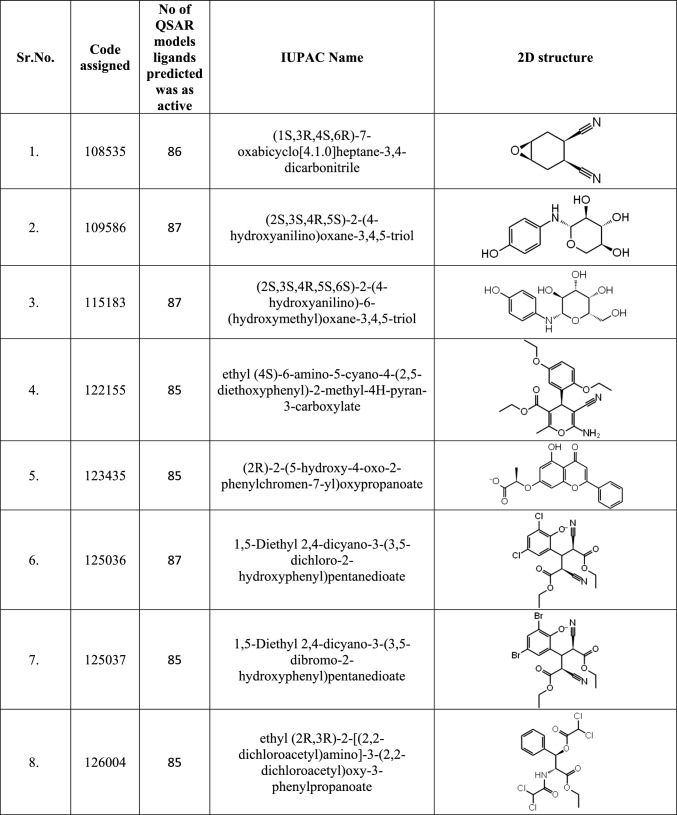

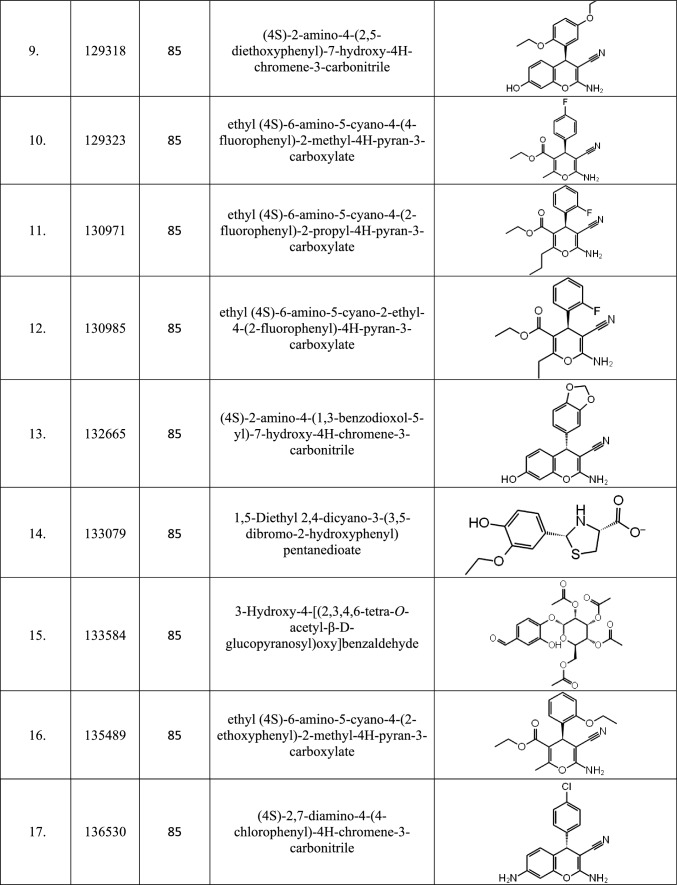

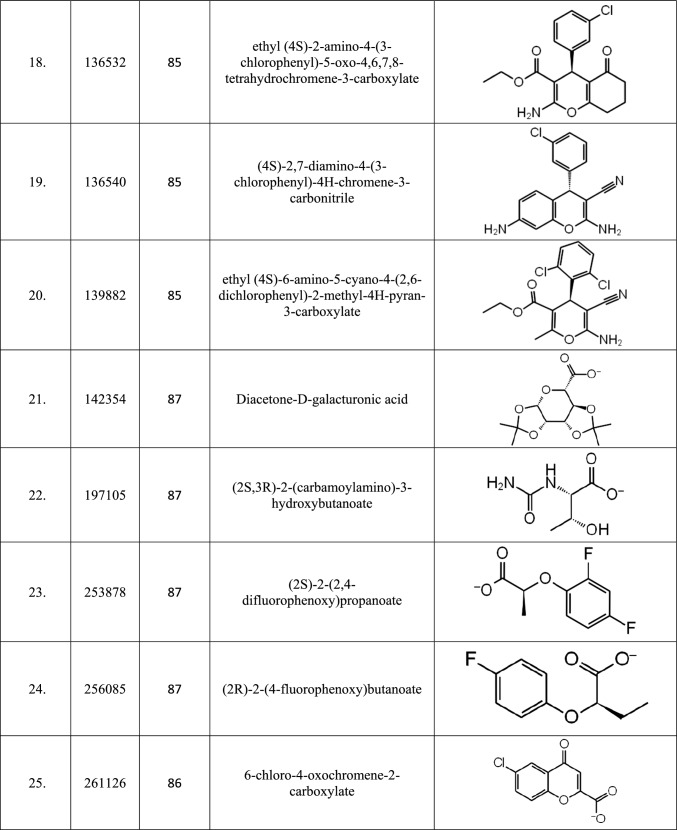
List of top twenty-five molecules selected based on Fingerprint-based 2D QSAR

### Shape-based virtual screening

The top twenty-five molecules obtained from fingerprint-based 2D QSAR were screened using known Akt activators, as discussed in Sect. 2.4. The shape sim score for the selected molecules is listed in Table 3. Of the twenty-five molecules, 133,584 showed the highest score in the five models obtained from quercetin-3-glucuronide (shape sim-0.3638), puerarin (shape sim-0.471), kaempferol-3-glucuronide (Shape sim-0.339), inositol 1,3,4,5-tetrakisphosphate (shape sim-0.421), and baicalin (shape sim-0.501), in the other two models chlorogenic acid (shape sim-0.285) and SC79 (shape sim-0.349) it had lower scores than the other molecules.

**Table 3:**
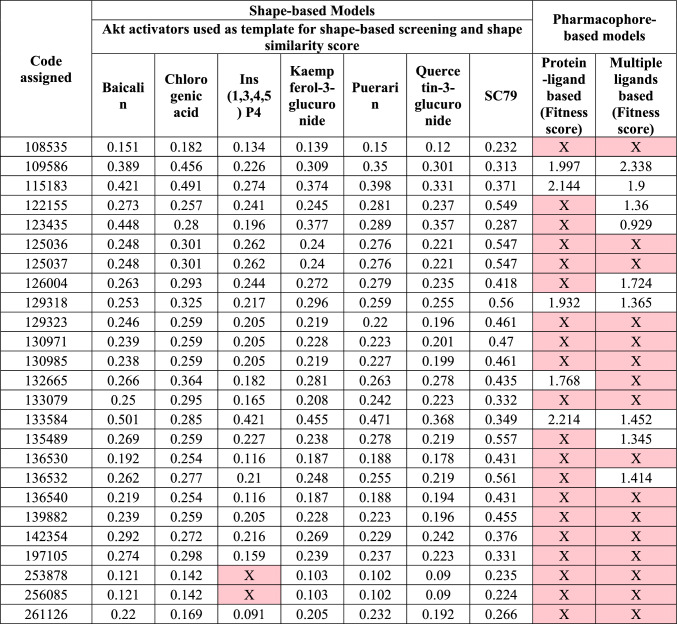
List of top 25 ligands selected after 2D QSAR based on the number of models in QSAR in which it was predicted as active ligand, Shape similarity score, fitness score obtained after screening with shape-based screening, and pharmacophore models, respectively

### Pharmacophore modeling and virtual screening

#### Protein–ligand complex-based pharmacophore

**Fig. 1:**
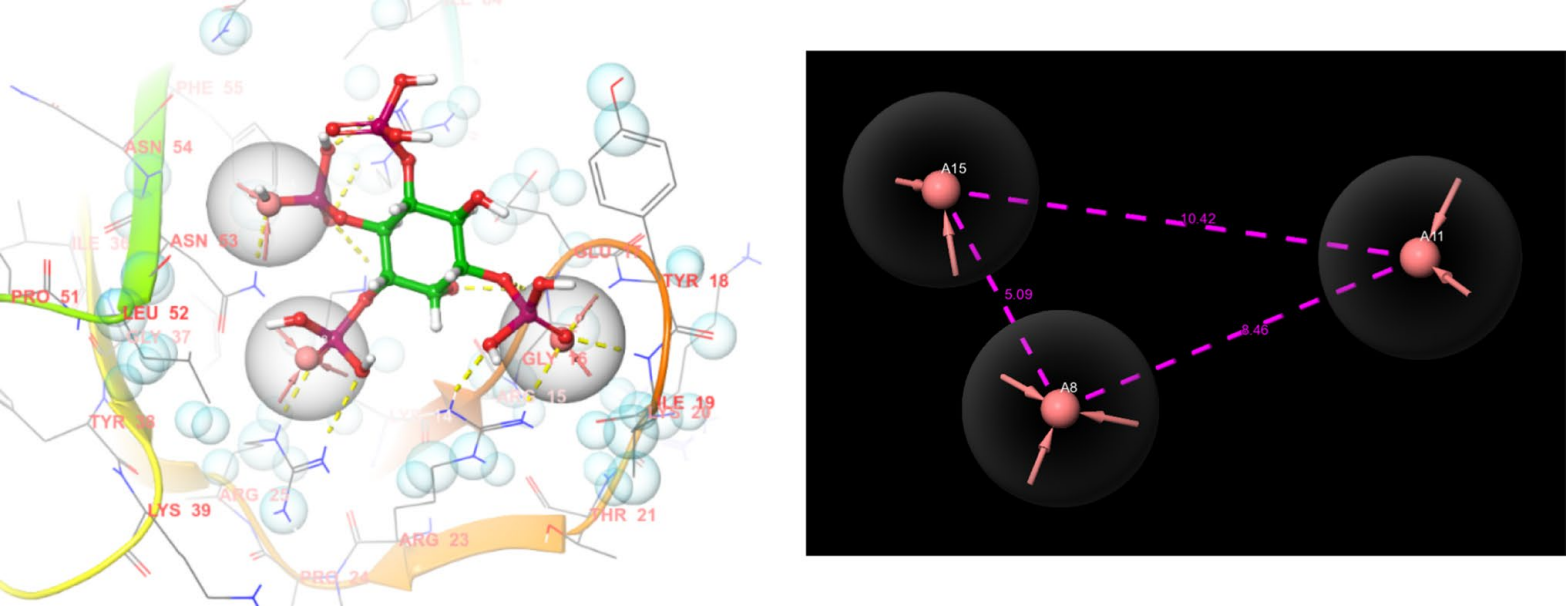
Protein–ligand complex-based pharmacophore model obtained by using 1UNQ

The model generated by this methodology has three acceptor groups, A8, A11, and A15, which are aligned with the three phosphate groups of inositol 1,3,4,5-tetrakisphosphate, which is the ligand bound to the PH domain of Akt1 (Figure 1). The A15 is 5 Å apart from A8, A15 is 10.42 Å apart from A11, and A11 is 8.46 Å apart from A8. Among the top twenty-five selected molecules, only five showed similarities with the generated model, of which 133584 (fitness score—2.214) showed the highest fitness score, which also showed the highest shape similarity score in the shape-based screening approach. Other molecules that emerged as hits in this model were 115183 (fitness score—2.144), 109586 (fitness score-1.997), 12931 (fitness score—1.932), and 132665 (fitness score—1.768). The fitness score of all twenty-five selected molecules has been tabulated in Table 3.

#### Multiple ligand-based pharmacophore

**Fig. 2:**
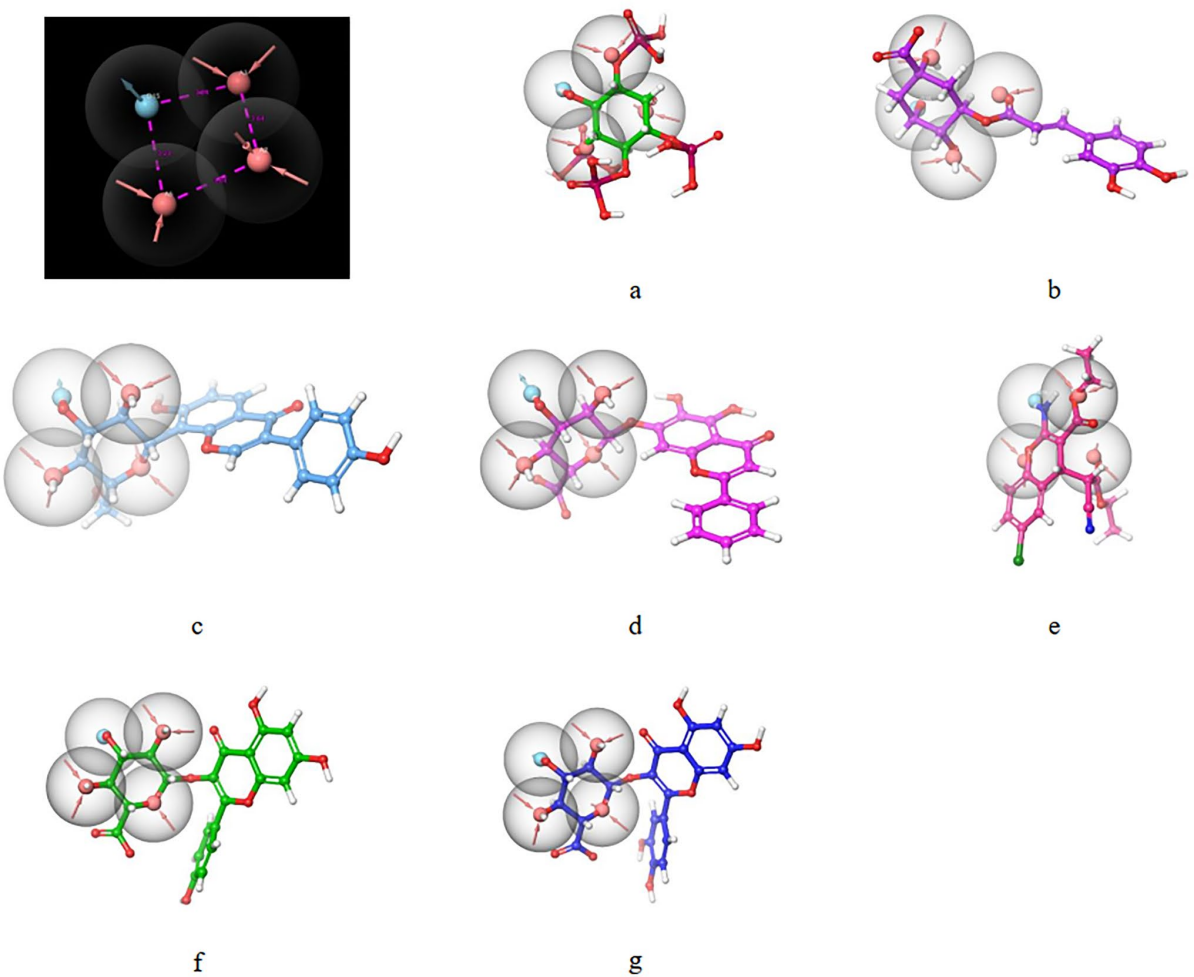
Multiple ligand-based pharmacophore model aligned with known activators like **a** inositol 1,3,4,5-tetrakisphosphate. **b** chlorogenic acid. **c** puerarin. **d** baicalin. **e** SC79. **f** kaempferol-3-glucuronide. and **g** quercetin-3-glucuronide respectively

Ten models were generated for multiple ligand-based pharmacophores (Table S2). Pharmacophore models were ranked according to the phase hypo score, among which AAAD_3, AAAD_5, and AAAD_9 had the highest phase hypo score of 1.17, but AAAD_5 had the highest BEDROC score of 0.85 among the three models; therefore, they were selected for screening. The model has three acceptor groups (A2, A3, and A5), and one donor group D15, as shown in Figure 2. The distance between the acceptor groups was less than 4 Å compared to the model obtained from Protein–ligand complex-based pharmacophore. It had a donor group aligned to the hydroxyl group in inositol 1,3,4,5-tetrakisphosphate. After the screening of the top twenty-five selected molecules, only nine molecules showed similarities to that of the model, out of which three molecules had fitness scores of more than 1.5, which are 109586 (fitness score—2.338), 115183 (fitness score—1.9), 126004 (fitness score—1.724). 109586 were positive in 86 models generated by fingerprint-based 2D QSAR and had a high score for protein–ligand complex-based pharmacophores.

#### Molecular docking and binding free energy calculation

**Fig. 3:**
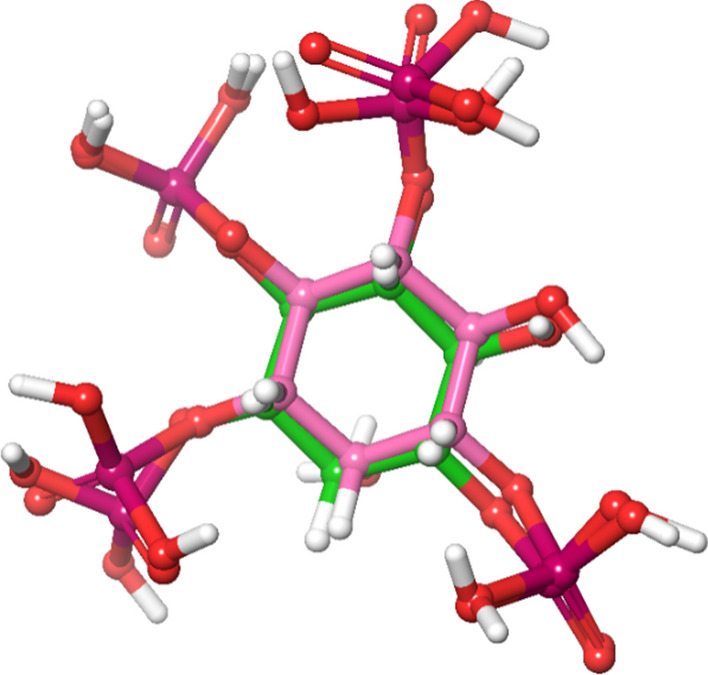
Superimposition of docked (green) and co-crystallized ligand (pink) pose of inositol 1,3,4,5-tetrakisphosphate of 1UNQ PBD for validating of docking protocol, the observed RMSD was 1.455

After performing extensive ligand-based approaches such as fingerprint-based 2D QSAR, shape-based screening, and pharmacophore-based virtual screening, the top twenty-five molecules were selected for molecular docking. The crystal structure of the full-domain Akt1 structure is required to perform structure-based approaches, which is currently unavailable in the active conformation. The only crystal structure of Akt1 in the active conformation is 1UNQ, which is the PH domain of Akt1 bound to inositol 1,3,4,5-tetrakisphosphate. Although the structure and ligand binding to the PH domain changes in the presence of other domains, owing to the lack of availability of the full-domain crystal structure, 1UNQ was selected for further structure-based approaches. Before docking the selected molecules, the generated grid was validated by redocking the inbound ligand, and the RMSD was found to be 1.455 (Fig. 3). Next, selected molecules and known activators, such as baicalin, puerarin, chlorogenic acid, kaemferol-3-glucuronide, and quercetin-3-glucuronide, were docked into the pocket. Chlorogenic acid showed the highest score (− 6.829 kcal/mol) among the known activators. Among the selected molecules, 197105 showed the highest score of − 4.978 kcal/mol, showing interactions with Lys 14, Arg 23, Arg 25, and Asn 53. Other molecules, such as 197105, 261126, 253878, 256085, and 123435, had binding affinities between − 4.876 and − 4.605 and interacted with key residues. The docking score, binding energy, and 2D interaction diagram of the known activators and selected molecules have been tabulated in Table S4 and Table 4, respectively.

**Table 4:**
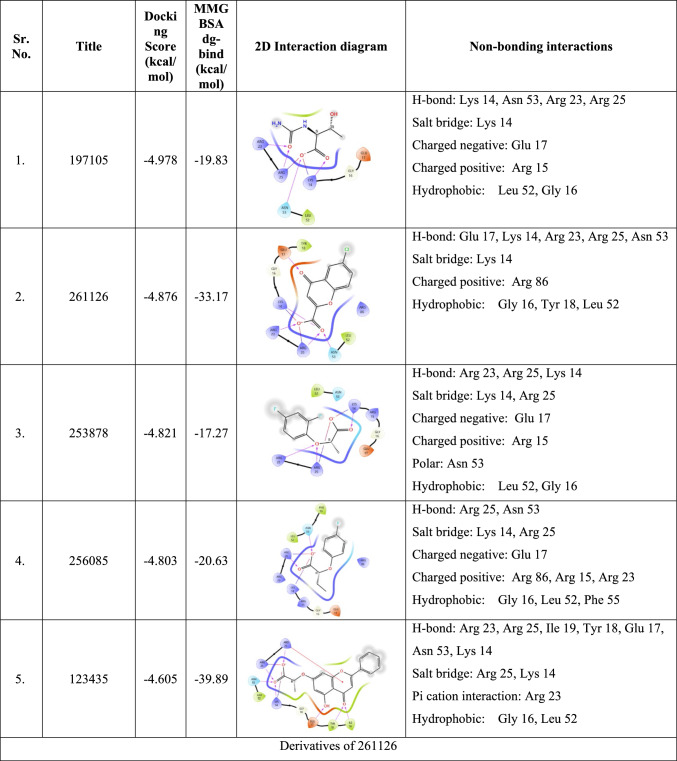

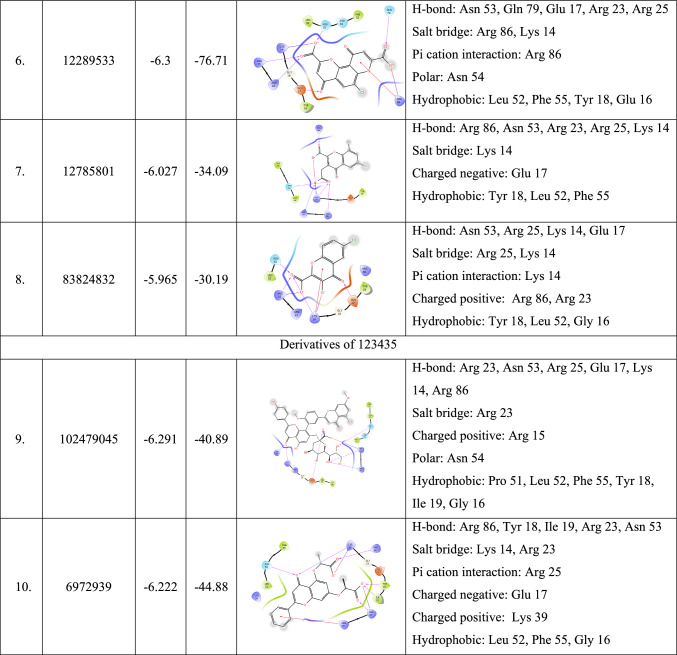
2D interaction diagrams of the top five lead molecules selected after molecular docking and derivatives of 261126, 123435 with a summary of docking score, binding energy, and all non-bonding interactions

Using Prime-MM-GBSA, the binding energies for the ligand were calculated, indicating the stability of the protein–ligand complex created during docking. The binding energy of the known activator ranged from − 40.96 to − 26.38 kcal/mol, among which quercetin 3-O-glucuronide had the highest binding energy of − 40.96 kcal/mol and SC79, the lowest of − 26.38 kcal/mol. For the selected molecule, 123,435 had the highest binding energy of − 39.89 kcal/mol, but in molecular docking, it showed the lowest dock score (− 4.605 kcal/mol) among the selected molecules.

#### Predicted ADME properties

The top five molecules, 123435, 197105, 253878, 256085, and 261126, were selected based on docking score, binding energy, and binding interaction with key residues and were subjected to ADME analysis using the Qikprop module. ADME properties included molecular weight, QPlogPo/w, QPlogS, rule of five, QPPCaco, percentage human oral absorption, CNS, and QPlogBB, tabulated in Table 4. All molecules have molecular weights within the acceptable range of 200–500 Daltons, QPlogPo/w, representing the partition between octanol and water, and values within the acceptable range of − 2 to 6.5. The predicted aqueous solubilities (QPlogS) were 6.5–0.5. None of the molecules violated any of these five rules. Except for 197105, all molecules had good permeability through QPPCaco and percentage of human oral absorption. All molecules had QPlogBB permeability within an acceptable range, but the predicted CNS activity was negative.

#### Molecular docking and binding free energy calculation of derivatives

After performing MD simulation of the top five molecules 123435, 197105, 253878, 256085, and 261126 selected after the ligand-based screening, molecular docking, MM-GBSA, and MD simulation were performed, and the results have been discussed in Sect. 3.7. After MD simulation, it was found that molecules 261126 and 123435 were more stable and had many interactions with key amino acid residues like Lys 14, Arg 23, Arg 25, Asn 53, and Arg 86. To further explore the effect of substitution on the scaffold of 261126 and 123435 in the ability to bind and form a stable complex with PH domain of Akt1 derivatives and to identify more potent Akt activator, derivatives were searched for 261126 and 123435 molecules by using PubChem database. The 261126 molecule had 664 derivatives, and the 123435 molecule had 55 derivatives. These derivatives were downloaded, prepared into the 3D structure, and docked in 1UNQ pocket, and the top five molecules based on docking score were selected (Table 4). The docking score of the derivatives was more than − 4.978 kcal/mol, which was the highest for molecules identified from the Asinex gold platinum database. The docking score for 12289533 was − 6.3 kcal/mol, the highest among the derivatives and selected molecules. The binding energy of the derivatives was also much better than that of the selected molecules from the database, the highest being − 76.71 kcal/mol for 12289533. The derivatives also have druggable properties, as shown in Table 5. Finally, five derivatives, 12289533, 12785801, 83824832, 102479045, and 6972939, were chosen for MD simulation.

**Table 5:** predicted ADME of the top five lead molecules by using various parameters like solubility, partition, toxicity, absorption, and druggability

ID assigned	Molecular weight	QPlogPo/w	QPlogS	Rule of five	QPPCaco	% Human oral absorption	CNS	QPlogBB
197105	162.145	− 1.005	0.257	0	6.008	35.001	− 2	− 1.63
261126	224.6	1.347	− 2.45	0	62.898	67.022	− 1	− 0.728
253878	202.157	2.747	− 2.479	0	405.519	89.709	− 1	− 0.096
256085	198.194	2.746	− 2.553	0	373.013	89.055	− 1	− 0.31
123435	326.305	3.486	− 4.608	0	46.493	77.2	− 2	− 1.471
Derivatives of 261,126								
12289533	336.642	0.34	− 2.776	0	0.678	25.912	− 2	− 2.183
12785801	317.082	1.869	− 3.232	0	4.981	50.37	− 2	− 1.221
83824832	240.599	1.422	− 2.905	0	31.329	62.047	− 2	− 1.075
Derivatives of 123,435								
102479045	816.725	1.997	− 4.521	3	0.145	0	− 2	− 4.949
6972939	398.368	2.939	− 4.004	0	5.888	57.933	− 2	− 1.828

#### Molecular dynamic simulation analysis

**Fig. 4:**
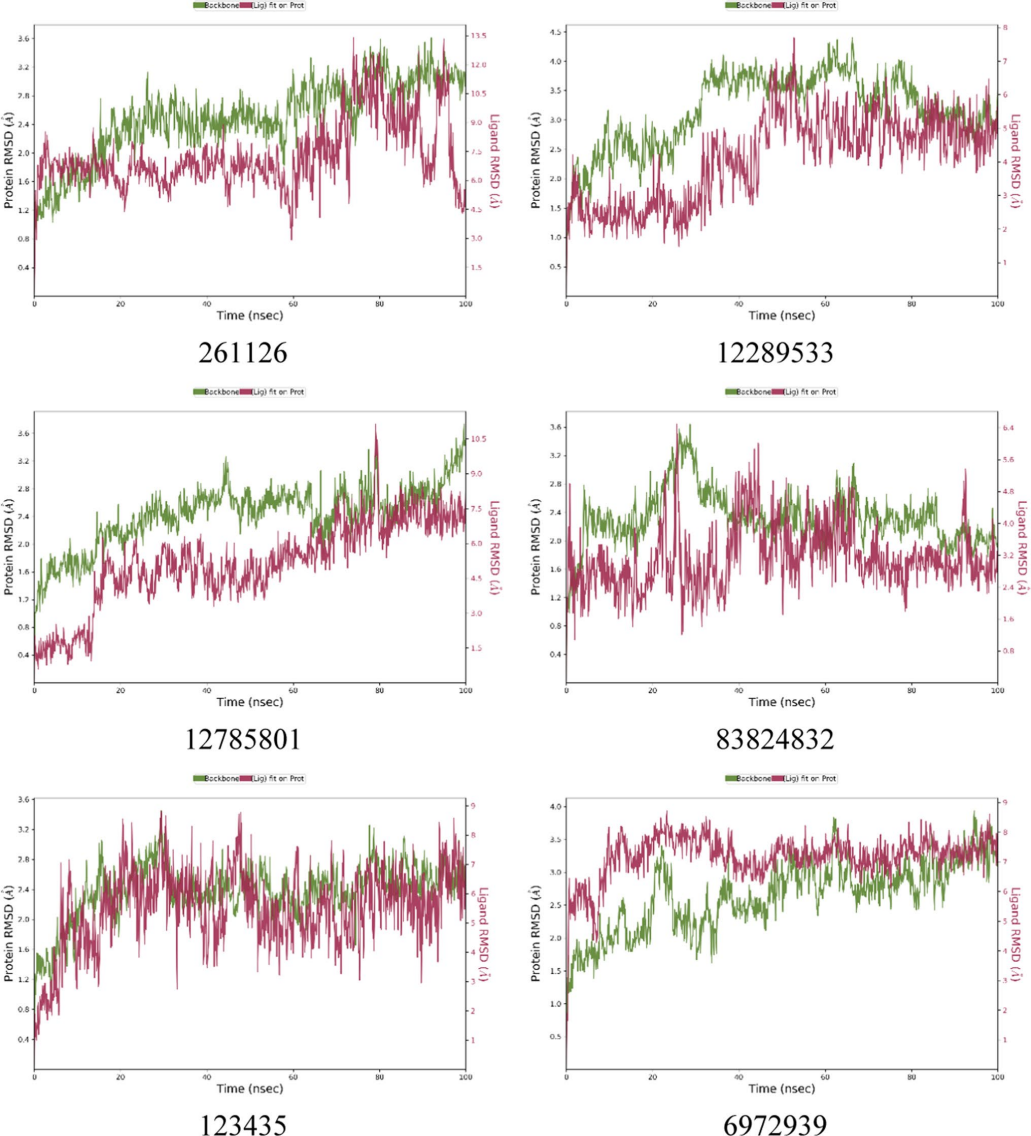
RMSD plots of protein and ligand complex after MD simulation for 100 ns

**Fig. 5:**
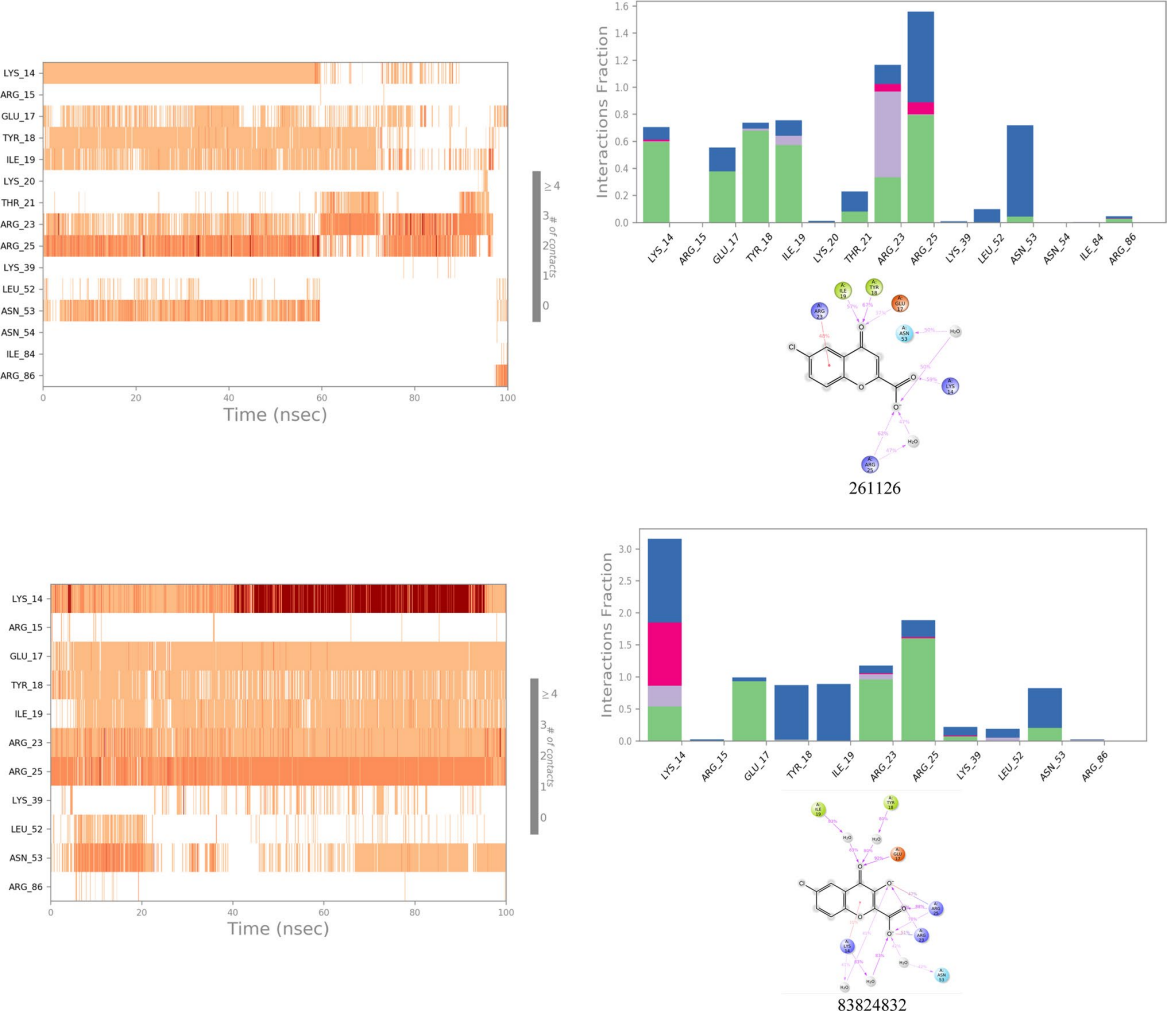

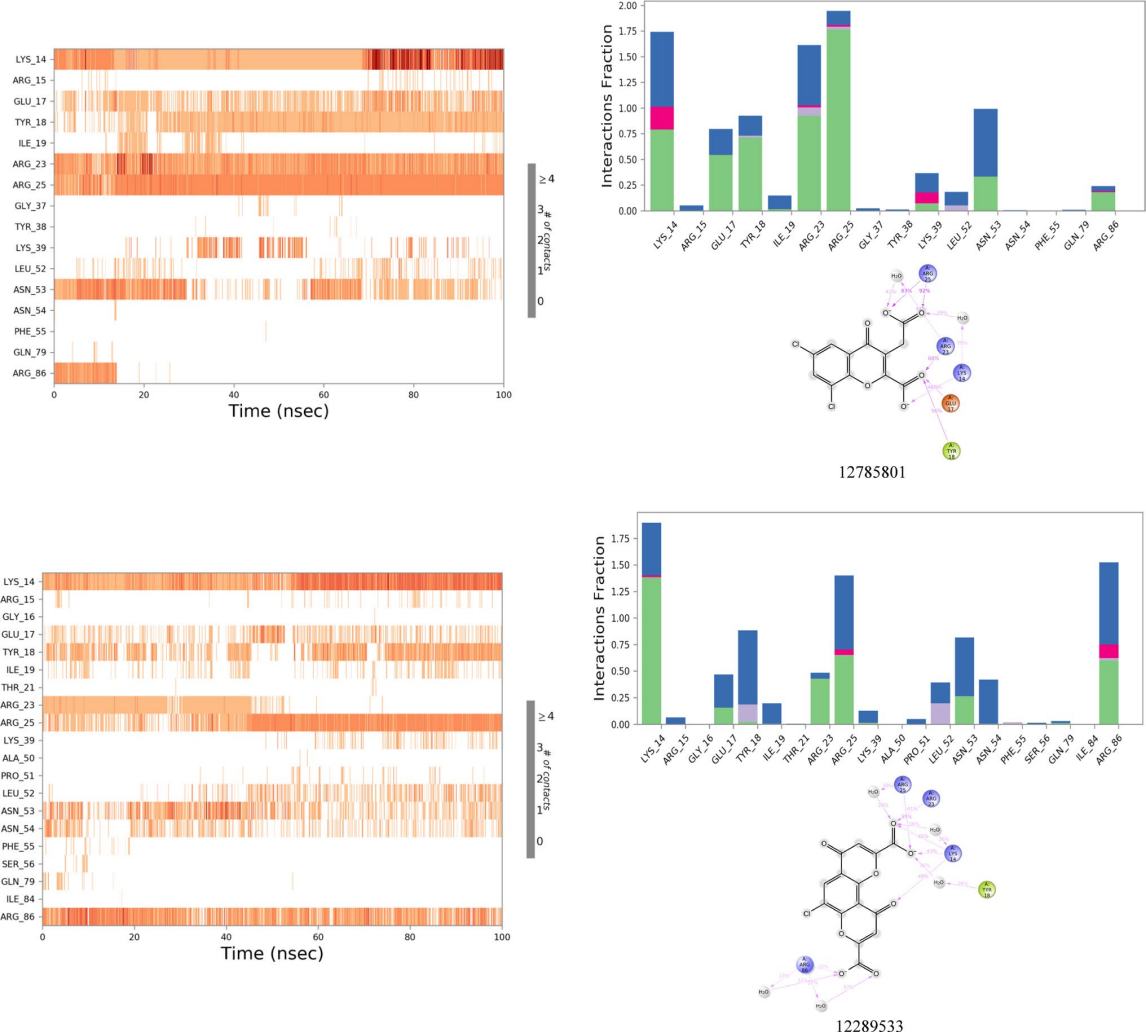

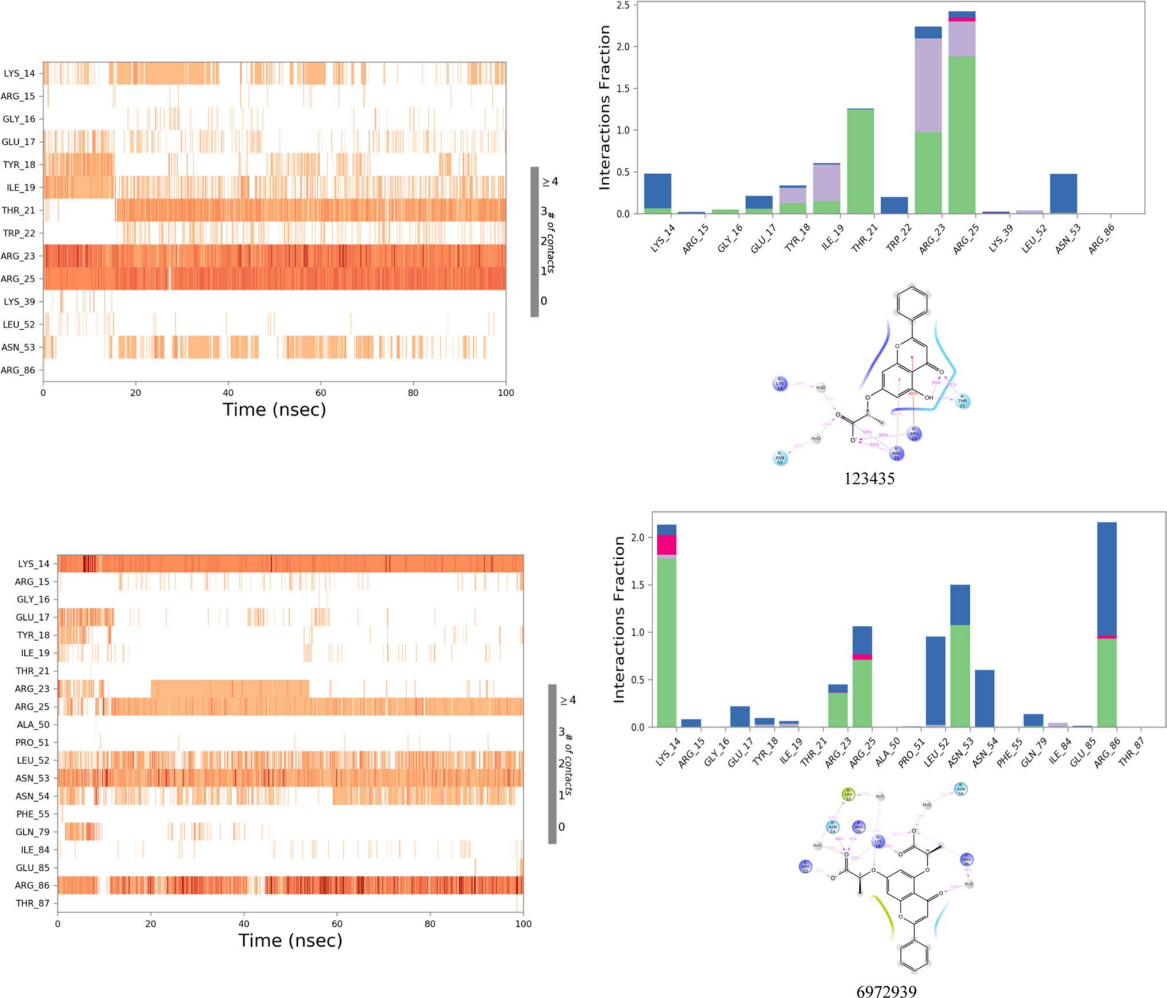
2D interaction diagram, protein–ligand contacts, and timeline representation of the interactions for 100 ns MD simulation in which green represents H-bonds, pink represents an Ionic bond, blue represents a water bridge, and gray represents hydrophobic interaction

The results of MD simulation have been illustrated for 123435 and 261126 and their derivatives like 12289,533, 12785801, 83824832, and 6972939 in Fig. 4 and Fig. 5 and discussed below. Other selected molecules like 197105, 253878, 256085, and 102479045 (derivative of 123435) were performed, but due to inadequate results, they have been not discussed in the manuscript but have been illustrated in Fig. 1S and Fig. 2S.

The protein 1UNQ–ligand 261,126 complex system was used for the simulation contained 6408 molecules of water, and the charge of the system was neutralized with two counter sodium ions. During the MD simulation, all the crucial XP interactions were observed along with the other interactions (Tyr 18 and Ile 19). However, loss of salt bridge with Lys 14 was observed, which was present in XP docking results. The system was stable for 60 ns with RMSD ranging from 4.5 to 7.5 Ǻ, after 60 ns RMSD fluctuated up to 12 Ǻ. Till 60 ns, a strong H-bond and water bridge interactions were retained with Lys 14, Arg 25, and Asn 53. However, the change in the RMSD after 60 ns might have occurred due to the loss of H-bond and water bridge interactions with three crucial amino residues, Lys 14, Arg 25, and Asn 53. Therefore, new derivatives of the 261,126 ligand were identified (83,824,832, 12,785,801, 12,289,533) and subjected to the MD simulation to acquire the most potent Akt activators.

Derivative of 261,126 ligand bearing 10-oxo-10H-pyrano[2,3-f]-8-carboxylate substitution (12289533) was selected for simulation study employing 1UNQ protein and 12289533 ligand complex. A fluctuation in the protein was observed till 60 ns, with the RMSD value ranging from 3 to 7.5 Ǻ. After 60 ns, the system stabilized, and the RMSD value of protein and ligand was found to be 3.5 and 5 Ǻ, respectively. A drift in the RMSD value till 60 ns was exhibited by the loss of strong H-bond and water bridge interactions with Arg 23, Arg 25, and Asn 53 residues. However, the system was stabilized after 60 ns due to H-bond and water bridge interactions between ligand and protein amino residue, Arg 25. Lys 14 and Arg 86 residues have contributed to the overall stability of the simulation system by retaining the H-bond and water bridge interactions throughout the simulation. Interaction with Arg 86 residue was pertained by the carboxylate group of the pyran ring system, which has not appeared in the case of the 261,126 ligand simulation system. Therefore, the system was relatively stable in comparison with the 261,126 ligand. However, it was subordinate in comparison to the 83,824,832 ligand. This might be due to the loss of H-bond and water bridge interaction with Arg 23, Arg 25, Asn 53, Glu 17, Tyr 18, and Ile 19 residues. The results of the 1UNQ-12289533 complex system revealed that the selected compound might show more potency as an Akt activator in line with 261126 ligand. Therefore, it should be selected for future studies to interpret its potency towards Akt activation.

Another derivative of 261,126 ligand bearing 3-methyl carboxylate substitution along with the 6,8-dichloro group on the 4-oxo-4H-chromene-2-carboxylate scaffold is entitled 12,785,801. The selected ligand was subjected to 100 ns simulation. The fluctuation in the RMSD was reported throughout the simulation. However, the system stabilized between 65 and 95 ns during the simulation period. After 95 ns RMSD value fluctuated up to 10.5 Ǻ. The alteration in the system might be reported due to the introduction of the methyl carboxylate group at the 3rd position, as it led to ligand fluctuation. Besides this, inconsistent H-bond and water bridge interactions of the ligand with Asn 53 and Arg 86 might lead to a significant drift in the RMSD value throughout the simulation.

Protein (1UNQ)–ligand complex of 83,824,832 derivatives of 261,126 was subjected to 100 ns simulation. The system comprises 6394 molecules of water and 986 molecules of heavy atoms. Three counter ions of sodium neutralized the charge of the system. The protein–ligand complex was stable throughout the simulation compared to the 261126 ligand with RMSD values 2.2 and 3.2 Ǻ, respectively. A slight drift was observed between 20 to 40 ns, but the RMSD deviation was less than 3 Ǻ which is well within the acceptable range. Stability to the 83824832 ligand system was contributed by introducing the 3-oxide group in the 261,126 ligand composed of the 4H-chromene-2-carboxylate scaffold, illustrated in Table S4. The introduction of the oxido group at the 3rd position helped to retain all the crucial H-bond and water bridge interactions with Lys 14, Arg 23, Arg 25, and Asn 53 residues. Besides, the system also maintained additional H-bond and water bridge interactions with Glu 17, Tyr 18, and Ile 19 residues which might also provide stability to the complex. The overall stability of the complex exhibited that 83824832 could act as a potential activator for Akt.

MD simulation study was conducted for the 123,435 ligand, which has phenyl, hydroxy, and propanoate groups substituted at the 2nd, 5th, and 7th positions of the 4-oxo-4H-chromene moiety. The protein–ligand complex system was stable throughout the simulation as per the RMSD plot. However, the RMSD value of the protein and ligand was found to be 3.8 and 7 Ǻ, respectively. A significant difference in the protein and ligand RMSD value was observed due to the introduction of the phenyl group at the 2nd position, which led to the contribution of high ligand fluctuation. Besides this, introducing the phenyl group also contributed to the loss of H-bond and water bridge interactions with crucial amino acid residues, such as Lys 14, Asn 53, and Arg 86.

Another simulation study was executed for 6,972,939, a derivative designed from the 123,435 ligand with 1UNQ protein to identify potent lead as an Akt activator. The ligand comprises 2-phenyl and 5,7-bis(oxy)dipropionate substitution on the 4-oxo-4H-chromene scaffold. In this simulation study, 1UNQ-6972939 complex system composed 6500 water molecules and 986 heavy atom molecules. Three counter sodium ions neutralized the charge of the complex. RMSD of protein and ligand was found to be 3.5 and 7.5 Ǻ. A drift in the RMSD value was observed from 0 to 50 ns, and the system was stabilized after 50 ns. The ligand has retained most of the crucial H-bond and water bridge interactions with Lys 14, Arg 23, Arg 25, Asn 53, and Arg 86 residues. However, a high RMSD value was reported for protein and ligand. The reason behind this might be the ligand fluctuation due to the introduction of the phenyl group at the 2nd position. The Phenyl group at the 2nd position led to a significant fluctuation in ligand RMSF value. Therefore, this ligand could be subjected to further studies with lead optimization to acquire potent Akt activators.

The simulation revealed that 83824832 and 12289533 could be potential lead compounds as Akt activators in the PI3K/Akt pathway. The study also predicted approximately the SAR (Structure–activity relationship) of the compounds bearing 4-oxo-4H-chromene moiety. The substitution of electron-withdrawing groups (EWG) might be favorable at the 2nd position, like the carboxylate group in 261126, 83824832, and 12289533, instead of the electron donating group (EDG) like phenyl in 123435 and 6972939 ligands for 4-oxo-4H-chromene moiety as Akt activator. Small EWD groups (like oxido groups) might be favorable for activity at 3rd position. Substitution of the halogen group at the 6th position might favor activity. However, substitution at the 5th and 7th positions might be unfavorable for the activity. Hence, the study will help to design the most potent Akt activators of the 4-oxo-4H-chromene scaffold using computational lead optimization approaches.

## Conclusion

The Akt pathway, which lies at the nexus of survival and cell death pathways, has been explored for many diseases like stroke, Alzheimer’s, Parkinson’s, and Diabetes. Still, only a few molecules like SC79 have been proven as Akt activators. SC79 has been studied for its effect on AD and stroke, which is relatively unstable in the aqueous environment, so it is impossible to develop as a drug molecule. Other known molecules like baicalin, chlorogenic acid, puerarin, kaempferol-3-glucuronide, and quercetin 3-glucuronide have been proven to bind to the PH domain of Akt1. However, they have not been studied thoroughly for their effect on Akt kinase and explored for other diseases as Akt activators.

In the present study, we have identified lead molecules that can act as Akt activators for the first time using computational tools like ligand-based and structure-based approaches. At first, a ligand-based approach like fingerprint-based 2D QSAR was used in which known Akt activators (like inositol 1,3,4,5-tetrakisphosphate, baicalin, chlorogenic acid, puerarin, kaempferol-3-glucuronide, and quercetin 3-glucuronide) and known inactive molecules were used to build 147 fingerprint-based 2D QSAR models. Of these, only 87 models predicted the active and inactive without error. Further, these 87 models predicted the activity of 305,168 molecules obtained from the Asinex gold platinum database. After that, the top twenty-five molecules found to be active using most of the fingerprint-based 2D QSAR models were selected. Shape-based models were built using known activators, pharmacophore models were built using 1UNQ PDB, and known activators were used to screen the top twenty-five molecules. After fingerprint-based 2D QSAR, shape-based screening, and pharmacophore structure-based approaches were used, docking with the PH domain of Akt1 (1UNQ PDB) was done. In which top five molecules were selected based on docking score and interaction with key amino acid residues like Lys 14, Arg 23, Arg 25, Asn 53, and Arg 86. The binding energy and ADME properties were predicted for the top five molecules. Further, an MD simulation was performed to prove that the selected top five lead molecules bind to the PH domain of Akt1. In MD simulation, two molecules, 123435 and 261126, were more stable and interacted with key residues longer than other selected molecules.

To study the effect of substitution on scaffold and to identify better Akt activator derivatives of 123435 and 261126 were searched on PubChem and Molecular docking, MM-GBSA and ADME prediction was performed. Finally, MD simulation of 12289533, 12785801, 83824832, 102479045, and 6972939 was performed in which 83824832 and 12289533 were found to be the most stable with showing interaction with key residues for a longer duration of time throughout the simulation. Therefore, based on the research work, we have identified two lead molecules, 83824832 and 12289533, which may act as Akt activators by binding to the PH domain and promoting phosphorylation of Akt in the cytoplasm. For further validation, *in-vitro* and *in-vivo* experiments should be performed to confirm its activity as Akt activators.

### Supplementary Information

Below is the link to the electronic supplementary material.

Supplementary file1 (DOCX 2664 KB)

## Declarations

### Preprint

The authors declare that the article is posted on the preprint server of research square (https://www.researchsquare.com/article/rs-2692696/v1).
